# Risk Factors for Six Types of Disability among the Older People in Thailand in 2002, 2007, and 2011

**DOI:** 10.1155/2016/6475029

**Published:** 2016-08-24

**Authors:** Pattaraporn Khongboon, Sathirakorn Pongpanich, Robert S. Chapman

**Affiliations:** ^1^College of Public Health Sciences, Chulalongkorn University, Pathumwan, Bangkok 10330, Thailand; ^2^Prince Mahidol Award Foundation under the Royal Patronage, Faculty of Medicine, Siriraj Hospital, Bangkoknoi, Bangkok 10700, Thailand

## Abstract

*Background*. There is an important need to characterize risk factors for disability in Thailand, in order to inform effective prevention and control strategies. This study investigated factors associated with risk of 6 types of disability in Thailand's ageing population in 2002, 2007, and 2011.* Methods*. Data came from the Cross-Sectional National Surveys of Older Persons in Thailand conducted by the National Statistical Office (NSO) in 2002, 2007, and 2011. Stratified two-stage sampling was employed. Interviews of 24,835, 30,427, and 34,173 elderly people aged 60 and above were conducted in the respective study years. Prevalence of disabilities was measured, and factors associated with disability risk were assessed with probability-weighted multiple logistic regression.* Results*. Disability prevalence decreased slightly over the study period. The characteristics with greatest positive impact on disability prevalence were not working over the past week (average impact: 61.2%), age (53.7% per decade), and suffering from one or more chronic illnesses (46.3%).* Conclusions*. The strong observed positive impact of not working on disability prevalence suggests that raising the mandatory retirement age might result in some reduction of disability risk. Also, the observed positive impact of living with others (versus alone) on disability risk was somewhat unexpected.

## 1. Introduction

The population of old people is rising exponentially in Thailand. According to The Foundation of Thai Gerontology Research and Development Institute, TGRI (2013) [1], the country has the second highest population of elderly folk in southeast Asia. The aged population includes persons who have lived for 60 years or more. It is projected that the aged population in Thailand will significantly rise to approximately 22 million by 2040. In 25 years, 33.5% of the entire Thailand population will be old people. People in this age group usually have the highest rate of disability and are often the most dependent.

In spite of the efforts made by the Thai government to enhance the quality of healthcare services for the older population, the prevalence of disparate access to services, inadequate quantity of healthcare resources (such as equipment, personnel, and finance), and lack of interinstitutional and intrainstitutional coordination among healthcare institutions still persists. In a bid to counter these problems, the Thai government commissioned a working group to make changes to the country's National Plan on Older Persons in 2009. The working group drew inspiration from the findings of an evaluation of this plan that had been previously conducted and recommended for the establishment of community-based long-term care institutions for the elderly. These institutions would offer medical and social care for the elderly in the comfort of their homes.

In the past, ADL scale was basically constructed for individuals suffering from stroke and has two items pertaining to climbing stairs and continence. A combination scale that assesses ADL, social involvement, and communication is known as the functional independence measure. An earlier step in disability intensity may be recognized as a limitation in instrumental activities of daily living (IADL). Such activities essentially involve cognitive functions and other basic actions such as shopping, telephoning, shifting from one place to another, and dealing with medicines and funds.

These days ADL and IADL are often used for measuring health in both clinical studies and community-based surveys of older group [2, 3]. The calculations regarding IADLs are asymmetrical across diverse cultures, even if the evaluations of ADLs have been normally regular among various countries and in varying time frame. All of those activities that are auxiliary in everyday life are different for every country as seen in the national surveys, due to cultural, geographical, and temporal deviations [4–8].

So, the Barthel Index has been modified for assessment of activity in daily living in Thai older people [9]. This ADL instrument comprises eating, grooming, transferring in house, toileting, walking, climbing stairs, dressing, bathing, control of urination, and control of defecation. In addition, Chula ADL Index (CAI) has been modified for assessment of instrumental activity in daily living, which includes walking outdoors, cleaning house, public transportation, cooking, and money exchange [9].

Also, the documents that reported the prevalence of disability in Thailand are rare. A study conducted by village health communicators in 1989 in a rural northern Thai community found that the overall prevalence rate of the disabled aged population was 6.3/1000 [10]. A study conducted in 1997 reported that 19% of participants aged 60 years and over have a long-term disability [11]. Later, the study in 2001 found that the prevalence rates of disability in females were higher than that of males among the elderly living in Central region [12].

With respect to international comparison, the occurrence of the ADL disability in a population aged 60 years and above was as much as 25.7% in Lebanon [13]. However, more recent studies carried out after 2008 showed that the ADL disability is not as widely prevalent, with a population comprising 65-year-old and older population in China showing a prevalence of around 14.9% [14], whereas in the United States it was about 18.4% [15].

Much effort has been invested in identifying the risk factors associated with how disability sets in, and the corresponding model proposed by Nagi (1976) [16] is a major source of input in this regard, constituting components related to pathology, functional impairments, functional limitations, and disability. Beyond any doubt factors like age and gender are linked with the functional limitation and complexity with ADLs and IADLs. People who are over 70 years of age are more prone to these problems in contrast to the individuals in their 60s [17]. Moreover, it was clearly expressed that females tend to show more difficulties than men when they are asked to perform a similar task [18–20]. Area of residence does not play a predominant role in alteration of function but it is generally believed that older people or rural areas show less functional restrictions than the residents of urban areas [21]. Therefore, it is demonstrated in the literature review that there are several elderly people suffering from functional disability, with an annual increase in this dependency rate.

Down the line, disabled individuals could be expected to require a certain amount of LTC services in formal or informal settings, within an institution, in the community or at home [22].

It is important to understand that the elderly have lots of different needs which are not adequately met, thereby forcing many of them to go about their daily lives with these additional challenges. Likewise, it is expected that the risks involving disability and long-term illnesses on the part of the elderly will see a steady and sharp rise because of the increase in people's longevity.

Nonetheless, no current studies have been conducted to look into the disability risk factors throughout the country beginning from the year 2002 to 2011.

In view of this, the study sought to determine the disability risk factors of six disabilities (eating, dressing, squatting, lifting a 5 kg object, ease of stair use, and being able to travel by bus) among Thai aged population in 2002, 2007, and 2011. For each of the disability items the question was phrased as “can you perform these activities by yourself?” with response categories “no, not all,” “yes, with aid,” or “yes, without aid.”

In so doing, the disability items were transformed prior to merging three datasets; then we defined disability as “older people who could not do the task” or “older people who could do it only with help.” In addition, we excluded the urination and defecation continence from our analysis, since it was considered as physical health in the survey.

## 2. Materials and Methods

### 2.1. Data Source and Sampling

The National Statistical Office (NSO) had conducted four cross-sectional surveys in 1994, 2002, 2007, and 2011. Unfortunately, the NSO does not allow using 1994 dataset, so there are three datasets. The prime goal of this survey is to form a database of demographic, socioeconomic, and health characteristics and living arrangements of people aged 50 years and over in Thailand can be represented nationally.

To obtain this goal, a stratified multistage sampling technique was done by the National Statistical Office in all 76 provinces of Thailand which were further classified based on administered classification into urban and rural areas. The blocks for municipal areas and villages for nonmunicipal areas were the main sampling units. The probability of selection depends on the number of households existing in a block or village. A total of 5,796 blocks/villages were chosen in 2002 and 2011, while 5,793 blocks/villages were chosen in 2007.

Secondly, the private households were sampled. There were 15 households including a person aged 50 years or older chosen systematically from each chosen block and for the nonmunicipal area; 12 households were chosen systematically from each village. In 2002 and 2011, 79,560 households were chosen for final sample and, in 2007, 79,542 households were chosen for final sample.

In this study we selected only people aged 60 and above so the interviews were conducted with 24,835, 30,427, and 34,173 people in the 2002, 2007, and 2011 surveys of elderly Thai people, respectively. It was covered by the survey of the elderly in Thailand with a variety of demographic, socioeconomic, and health characteristics and management of people. Disability indicator in old age was the capability of doing activities on daily basis which is associated with the need for personal assistance.

Since the surveys have almost the identical questions on doing activities, then the availability of three cross-sectional datasets potentially presents the capability to quantify the differences of disability between three points of time while caution will require to be exercised.

### 2.2. Data Management

Data management will be undertaken to ensure database integrity and confidentiality. The following were approached.

#### 2.2.1. Data Cleaning

This part deals with detecting and removing errors and inconsistencies from data in order to improve the quality of data. Also, missing data were coded as system-missing in SPSS.

#### 2.2.2. Data Extraction

This part was employed in order to determine whether the formatting of the variables needs to be changed. Consistency in setting up response category values such as yes and no is one way to make the dataset more user friendly. The original response category values of variables of each study are shown in Table 1.

#### 2.2.3. Data Transformation

The variables were recoded to make the data fit with the purpose of the study. To do so we identified variables that were present in three datasets and recoded them. The identifier variable assures that the values of the same individual are on the same line. At this step, the dependent variables were recoded to dummy variable (see the new variables list in Table 2).

Persons who could do the task without help were considered not to have the disability. Persons who could not do the task or could do it only with help were considered to have the disability.

#### 2.2.4. Data Combination

The purpose is to have a longitudinal file which allows the understanding of changes, so we combined files from different years in order to run a regression utilizing year indicators.

### 2.3. Study Variables

#### 2.3.1. Dependent Variables

In the 2002 survey, eight items related to self-care were questioned (feeding, dressing, bathing plus toileting, squatting, lifting 5 kg objects, walking 1 kilometer, stair climbing: 2-3 steps, and transferring by bus or boat alone). In 2007 survey, nine items related to self-ability were questioned including eating, dressing, bathing/toileting/toothbrushing, squatting, lifting 5 kg object, walking 200–300 meters, stair climbing: 2-3 steps, getting out by bus or boat alone, and money management. In 2011 survey, fourteen items were queried (feeding, putting on clothes, taking a bath, washing face/brushing teeth, toileting/cleaning oneself after urinating/defecating, shaving/combing, putting on shoes, squatting, lifting 5 kg object, walking 200–300 meters, stair climbing: 2-3 steps, taking a bus or boat alone, money management, and taking medicine correctly).

Since three surveys have six identical questions on doing activities, there are six activities in this study that determine disability status: eating, dressing, squatting, lifting a 5 kg object, ease of stair use, and being able to travel by bus or boat. Survey participants in all three years (2002, 2007, and 2011) specified for each task whether they

1. could not do the task at all;
2. could do the task with help;
3. could do the task without help.

Before merging the data, we recoded these 3 responses into a dummy variable. Persons who could do the task without help were considered not to have the disability. Persons who could not do the task or could do it only with help were considered to have the disability.

#### 2.3.2. Independent Variables

The following sixteen independent variables were transformed to dummy variables and considered in multiple logistic regression analysis as follows: (1) age in years, (2) gender, (3) education, (4) income, (5) married but not living as a couple (unmarried is reference), (6) married and living as a couple (unmarried is reference), (7) working status in the past 7 days, (8) any of five chronic diseases, (9) area (0 = urban; 1 = rural), (10) year 2007 (year 2002 is reference), (11) year 2011 (year 2002 is reference), (12) living in Bangkok, (13) the Central region (excluding Bangkok), (14) the Northeast region, (15) the North region (the South region is reference for all regions), and (16) living arrangements were self-reported during an interview according to living alone = 0 and living with others = 1.

### 2.4. Data Analysis

Descriptive statistics were used to summarize characteristics of study participants. Logistic regression analysis was used to assess the extent to which the selected independent variables explain the disability of the elderly in Thailand. Confidence intervals (CIs) were calculated at the 95% level to estimate statistical significance. Sample probability weights were applied to data for each year. Statistical analysis was conducted using IBM-SPSS version 18.

In addition to odds ratios, the modeled absolute prevalence of each disability was calculated with and without each independent variable, at the mean values of all other independent variables. For each independent variable, the resulting difference in modeled prevalence was calculated as a proportion of weighted prevalence of that disability. This procedure gave a measure of the impact of each independent variable on each disability (e.g., the modeled prevalence of difficulty lifting 5 kg, in persons who had and had not worked during the past 7 days, and at the mean levels of all other independent variables was 32.8% and 15.6%, respectively, for a modeled prevalence difference of 17.2%. This difference constituted a proportion of 0.560 of the weighted lifting disability prevalence of 30.7% (0.172/0.307 = 0.560)). Then, for each independent variable, these proportions were averaged over all 6 disabilities. To enable comparison of the relative impacts of the independent variables, proportions for the independent variables were arranged in descending order of this average proportion. (This approach is somewhat similar to calculating and comparing modeled population-attributable risk fractions for the independent variables, except that it does not take their prevalence into account. Thus this approach may be viewed as an assessment of “absolute impact” of the risk factors, regardless of their frequency in the study population.) Also, this approach adds the benefit of considering absolute prevalence differences associated with risk factors and not merely odds ratios, which differ substantially at different baseline prevalence rates of studied outcomes and which can therefore be misleading.

### 2.5. Ethics Statement

As indicated above, the analyses reported here employed secondary data, which were originally collected by Thailand's National Statistical Office (NSO) in its nationwide surveys of 2002, 2007, and 2011. The NSO obtained written consent from all participants in these surveys. Completed consent forms are on file at the NSO. Additionally, the analyses reported here were approved by the Ethics Review Committee for Research Involving Human Research Subjects, Health Sciences Group, Chulalongkorn University, Certificate of Approval number 031/2015.

## 3. Results

### 3.1. Characteristics of the Study Population

The total numbers of the elderly with the people aged 60 and above were about 5,969,030 people in 2002, 7,020,959 people in 2007, and 8,266,304 people in 2011. The mean age of all participants was 68.6, 69.0, and 69.2 years in the 2002, 2007, and 2011 surveys, respectively. Females represented the majority of the population in all three surveys. More than 90% of the elderly in the samples lived with others, decreasing slightly but continually from 93.7 in 2002 to 92.3 in 2007 and to 91.4 in 2011. The percentage of elderly people who had at least one chronic disease increased from 29.6 in 2002 to 40.3 in 2007 and to 43.8 in 2011 (Table 3).

The number of elderly people who had not attended school decreased from 20.6 in 2002 to 16.4 in 2007 and to 11.8 in 2011. Approximately 30% lived in the Northeast region, which had the highest proportion of elderly people of all regions in Thailand.

### 3.2. Prevalence of Disabilities in the Three Study Years

**Figure 1:**
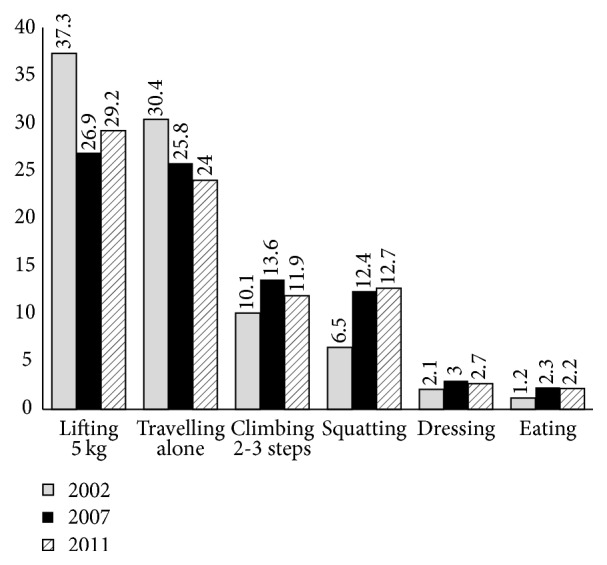
Weighted prevalence (percent) of the six studied disabilities by year.

Figure 1 and Table 3 show the prevalence of the six limiting activities by years. It was found that the prevalence of difficulty in lifting 5 kg decreased from 37.4% in 2002 to 27.0% in 2007 and increased slightly to 29.2% in 2011. Prevalence of difficulty in transportation declined somewhat, from 30.5% in 2002 to 25.8% and 24.0% in 2007 and 2011, respectively. The percentage of the elderly in 2002 that reported difficulty to step up 2-3 stairs was 10.1% which was increased to 13.6% in 2007 and it witnessed a bit decrease to 11.9% in 2011. For difficulty in squatting, the prevalence was 6.5%, 12.4%, and 12.7% in 2002, 2007, and 2011, respectively. The prevalence of the elderly who reported being difficulty in dressing was 2.1% in 2002 which increased to 3.0% in 2007 and then slightly decreased to 2.7% in 2011. The prevalence of elderly persons who had eating limitation was 1.2% in 2002, 2.3% in 2007, and 2.2% in 2011. Overall, prevalence of lifting-related and travel-related disabilities was high, prevalence of squatting- and stair climbing-related disabilities was intermediate, and prevalence of dressing- and eating-related disabilities was low.

Over all three surveys, the disability with highest prevalence activity that was most difficult for the sample population was lifting 5 kg (30.7), followed by traveling alone by bus or boat (26.4). Twelve percent of elderly people had difficulty climbing 2-3 stairs, a proportion slightly higher than that for squatting (10.9).

### 3.3. Logistic Regression of Risk Factors over All Three Survey Years

Table 4 demonstrated the associations of subject characteristics with disability risk. As expected, age indicated the highest risk for difficulty with dressing (OR = 1.09, 95% CI: 1.09–1.10), eating (OR = 1.09, 95% CI: 1.08–1.10), using stairs (OR = 1.10, 95% CI: 1.09–1.10), traveling alone (OR = 1.12, 95% CI: 1.12–1.13), lifting 5 kg (OR = 1.10, 95% CI: 1.09–1.10), squatting (OR = 1.08, 95% CI: 1.08–1.09), and any disability (OR = 1.11, 95% CI: 1.10–1.11) at *p* < 0.001.

For education level, no education indicated highest risk for difficulty with dressing (OR = 1.34, 95% CI: 1.20–1.49), eating (OR = 1.59, 95% CI: 1.41–1.79), using stairs (OR = 1.21, 95% CI: 1.14–1.28), traveling alone (OR = 1.42, 95% CI: 1.36–1.49), lifting 5 kg (OR = 1.27, 95% CI: 1.21–1.33), squatting (OR = 1.13, 95% CI: 1.06–1.20), and any disability (OR = 1.39, 95% CI: 1.32–1.45) at *p* < 0.001. Income inadequacy showed highest risk for difficulty with dressing (OR = 1.14, 95% CI: 1.29–1.54), eating (OR = 1.29, 95% CI: 1.16–1.43), using stairs (OR = 1.35, 95% CI: 1.29–1.41), traveling alone (OR = 1.28, 95% CI: 1.23-1.33), lifting 5 kg (OR = 1.09, 95% CI: 1.05–1.13), squatting (OR = 1.26, 95% CI: 1.20–1.32), and any disability (OR = 1.20, 95% CI: 1.16–1.40) at *p* < 0.001. Living with others indicated highest risk for difficulty with dressing (OR = 2.79, 95% CI: 2.21–3.52), eating (OR = 1.96, 95% CI: 1.54–2.51), using stairs (OR = 1.38, 95% CI: 1.27–1.51), traveling alone (OR = 1.18, 95% CI: 1.10–1.26), lifting 5 kg (OR = 1.10, 95% CI: 1.03–1.17), squatting (OR = 1.45, 95% CI: 1.32–1.59), and any disability (OR = 1.12, 95% CI: 1.05–1.19) at *p* < 0.001.

Having at least one chronic disease (heart problems, diabetes, hypertension, cancer, or stroke) had a highly significant association with dressing (OR = 3.41, 95% CI: 3.10–3.76), eating (OR = 3.19, 95% CI: 2.85–3.56), using stairs (OR = 2.16, 95% CI: 2.06–2.26), traveling alone (OR = 1.85, 95% CI: 1.78–1.92), lifting 5 kg (OR = 1.70, 95% CI: 1.64–1.76), squatting (OR = 2.07, 95% CI: 1.97–2.17), and any disability (OR = 1.77, 95% CI: 1.71–1.83) at *p* < 0.001. Being female had a negative association with difficulty in eating and dressing and a positive association with squatting, lifting 5 kg, using stairs, traveling by bus, and any disability. In terms of marital status, those who were married and lived with a spouse showed better ability to perform daily activities than their counterparts who were not married (divorced, widowed, and separated). Those who lived in a rural residence had a higher likelihood of having difficulty performing with any disability and traveling alone by bus/boat than those in an urban residence, but they were less likely to have difficulty eating, dressing, squatting, lifting 5 kg, and using stairs.

Interestingly, being unemployed seven days prior to interview indicated the highest risk for difficulty with dressing (OR = 6.03, 95% CI: 4.88–7.45), eating (OR = 5.15, 95% CI: 4.10–6.47), using stairs (OR = 4.04, 95% CI: 3.73–4.37), traveling alone by bus (OR = 3.28, 95% CI: 3.12–3.44), lifting 5 kg (OR = 2.64, 95% CI: 2.53–2.76), having any disability (OR = 2.64, 95% CI: 2.54–2.75), and squatting (OR = 2.61, 95% CI: 2.44–2.80) at *p* < 0.001.

**Figure 2:**
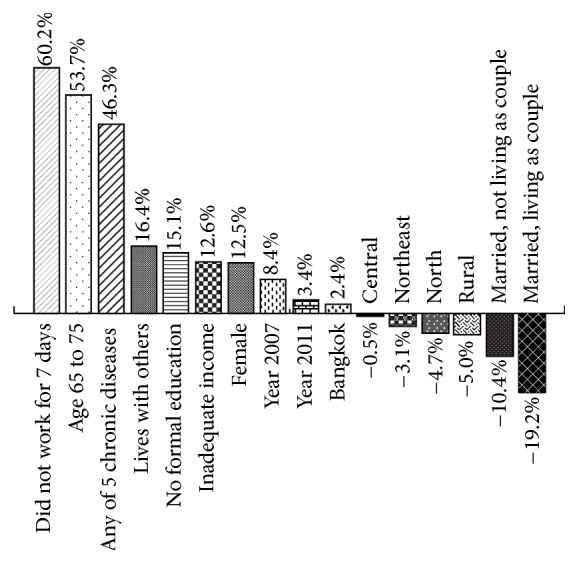
Proportional impacts of modeled independent variables on prevalence of the 6 disabilities studied.

Proportional impacts of independent variables on disability prevalence are presented in Figure 2 and Table 5. On average, the characteristics with the greatest adverse impact on disability prevalence were not working in the past 7 days (average impact: 61.2%), age per 10 years (53.7%), and presence of one or more chronic diseases (46.3%). Living with others, lack of education, and lower income were associated with moderate positive proportional impacts on disability prevalence (average impacts: 16.4%, 15.1%, and 12.6%, resp.). Female gender was associated with an overall positive impact but exhibited considerable variability across disabilities (average 12.5%, range 37.6% to −12.2%). Proportional impacts of study year varied widely across disabilities. Impacts of region were generally modest; there was appreciable variability across disabilities. Living in a rural area was generally associated with lower disability risk than living in an urban area. Being married was consistently associated with lower risk than being unmarried, especially for married participants who lived together as couples.

## 4. Discussion

### 4.1. Main Results

The levels of difficulty experienced by those surveyed decreased between the 2002 and 2007 surveys. The sole category in which the level of difficulty decreased between 2002 and 2007 and increased between 2007 and 2011 was the ability to travel alone by bus or boat. In one to two disabilities, the level of difficulty was reported to decline in 2007 and increase mildly in 2011. In more than three disabilities, the opposite was the case, with the level of difficulty rising in 2007 and dropping in 2011. Across all six activities, the overall level of difficulty decreased slightly. As it is not necessarily the case that all surveys were completed by the concerned parties themselves, there may be slight inconsistencies found with regard to the levels of difficulty observed in the results. The reasons for these trends are complex and include shifts in socioeconomic status of the elder population, in the distribution of underlying conditions and limitations in capacity that may be related to use of medical treatments, and in the uptake of assistive and other convenience technologies.

This study has shown that the factors most associated with disability are old age, being female, lack of education, low income, cohabitation, unemployment seven days prior to interview, having at least one chronic disease (hypertension, heart problems, diabetes, strokes, or cancer), and living in a rural setting. It has already been demonstrated in past studies that categories such as age, gender, and socioeconomic status may be linked to physical disability in the elderly [23, 24]. This study connects the factors of age, income, and marital status to the issue. Age is the most commonly noted associative factor with physical disability.

### 4.2. Relation to Other Research Findings

There are variations in occurrence of the ADL disability in different studies. It is not easy to compare the different studies because a variety of the ADL measurements and age groups have been used. In Malaysia, people over 60 had an ADL disability prevalence of 10.6% [25]. The uniqueness of the studies lies in the fact that the sample used in Malaysia was younger (mean age: 69.0) than that of the U.S. and Chinese population samples (74.5 and 75.1). Ofstedal et al. (2007) [26] show that prevalence of ADL and IADL limitations varies across Asian countries and trend was increasing. The prevalence of ADL disability in Singapore was 3.9% (1999), in Beijing it was 4.7% (1997), in Indonesia it was 6.5% (1997), in Taiwan it was 9.2%, and in Philippines it was 14.7% (2000). IADL restrictions in Singapore were 17.2% (1999), in Beijing they were 17.7% (1997), and they were 25.1% in Taiwan and 27.7% in Philippines (2000).

At present, there is a higher burden of dependency in Sub-Saharan African region, which has the dependency region of almost 20%. It is expected that there will be a high increase in this ratio in Sub-Saharan Africa, Latin America, and Asia. It was estimated in this study that there will be a higher increase in the dependency ratio in China (16%) and India (14%) in the next few years, along with the population increase. The study ended on the note that several countries are going to face the burden of rising number of dependent people and a lot more significance needs to be awarded to disability prevention [27].

The physical and mental health issues of the elderly population living in a rural region of Sepand in Malaysia were examined in a cross-sectional study. The random sampling technique was used to choose 5 out of 9 villages. There were 223 participants in the study aged more than 60 years. Prestructured questionnaires were used for interviewing the elderly in the villages, including geriatric depression scale, Barthel's Index, and elderly cognitive assessment questionnaire. The existence of physical health problems like chronic illness and functional dependence was found to be 60.1% and 17.5%. It was further found that the existence of physical reliance in at least one ADL was 17.5% and it was 17.1% for one or more ADLs. The most dependent activities for these people were bathing and toileting [28].

Basic ADL disability and functional restrictive rates among elderly population in America were examined in a study spanning from 2000 to 2005 so as to ascertain if there was an increase or decrease in the percentages of basic ADL disabilities and functional restrictions among the community residents and the institutionalized elderly. The yearly percentages of prevalence of basic ADL disabilities and functional restrictions were computed using data from American Community Survey and National Nursing Home Survey. This data was then used in regression lines to evaluate the changing trends with time. It was observed that there was a 9% increase in the percentages of basic activities of daily living (BADL) disabilities among the elderly in the community aged 65 and over. The institutionalized elderly ADL disability rates were found to be consistent among males but showed an increase for females in the years 2000 to 2005. It was henceforth deduced in this study that this growth in ADL disability with time among the elderly population greatly affects the healthcare mechanism [29].

Even if there is a huge range of disparity in the way surveys have questions regarding ADL functioning, there is some agreement among all of these surveys related to what activities of everyday life should be incorporated. According to Guralnik et al. [30], the calculations might differ merely due to the reason that the participants have a different understanding of the question seeing as the self-report instruments do not include distinct meaning for the calculated activity or plausible answer categories. As stated by M. W. Linn and B. S. Linn [31], the criteria of evaluation of disability by individuals can be affected by language, education, and culture. The factors like time period of disability, the kind of help that was given, and what range of complexity they experience while performing every ADL vary in surveys. Result of our study cannot be straight compared with these results but we can generalize that any disability is elevated along with the Thai elderly.

In terms of gender, ADL disability variations that indicate disability prevalence are more common among females compared to males [25, 32]. A study undertaken in Lebanon revealed that higher disability existed in elderly women (31.3%) than among elderly men (18.7%) [13]. Another study in Spain on people aged 65 indicated that the ADL disability prevalence among females was 15.8% and for males it was 10.6% [32]. Such differences may be attributed partially to a higher mortality rate among males and a higher ADL disability prevalence in females [33].

Few studies have examined differences in health and ADL disability based on residence (urban versus rural). Most studies emanate from Asia. The studies have indicated that greater ADL disability prevalence and poor health are found among aged rural dwellers [34, 35]. Another study in Australia and Canada established that 18-year-old adults from rural areas had poor health. It was discovered that a correlation existed between the health of over 18-year-old adults and living in rural areas, particularly with regard to poor health [36]. Another study in Finland found similar outcomes for 52-year-old adults [37]. Additionally, adults living in rural areas exhibited an unhealthy way of life, for instance, smoking, overweight, unhealthy eating habits, and less physical activity.

With regard to disability, income, rather than education, appeared related in the current study. Some past studies corroborate the finding [24, 38], whereas others state that income has no role in the elderly disability [39]. However, low-income individuals are not likely to seek proper healthcare.

Interestingly, this study found that aged individuals living with others had a higher risk of disability, an outcome that is consistent with Tsai et al.'s study [40]. It is possible that individuals living alone must complete chores on their own whereas those who live with others have assistance as aged couples assist each other when functional ability wanes. The study may not confirm the relation between living together and disability. The older people might invite another person to come and stay with them when they become disabled. Further research should be undertaken to explain this finding, even though many studies indicate that low economic status and loneliness increase the risk of disability among the older people.

The reason for the strong association between the risk of disability and the status of not working in seven days before the interview is not clear. The high probability may be due to the difficulty of defining unemployment, as it may include people who are not working because of a disability, those who are looking for work or are on temporary sick leave, the retired, home makers, and people whose spouse and/or children do not allow them to work. Thus, this issue must be further explored in depth.

### 4.3. Strengths and Weaknesses

The main strength point of the study is to provide description of aged individuals and to create a framework of forming practices that offer a better insight on Thai ageing. Different disability limitations and risks in Thailand in 2002, 2007, and 2011 are examined thoroughly in the study and the findings can be applied to the broader Thai population.

This study did not cover all disabilities. Moreover, the surveys depended on the respondents' personal description of circumstances. Thus, the possibility of inaccurate data could not be ruled out, particularly if a family member or friend participated in the reporting.

This study did not consider health behaviour variables, such as drinking clean water, smoking, and exercise, because these behaviours were not queried consistently in the 3 survey years. However, evidence from literature reveals that exercise and physical activities positively affect ADL and physical functioning among the aged. This may reduce the risk of facing an ADL disability. A study in the U.S. found a relationship between lower incidents of disability and higher rates of physical activity [41]. The current study omitted self-rated health as an independent variable because many individuals can rate their health basing on their past health. Finally, since the study was cross-sectional with regard to design, it is possible that determining the causality was not achieved.

### 4.4. Policy Recommendations

Because chronic illnesses relate strongly to disability, health prevention mechanisms of reducing later life disability should be promoted. Another option for reducing later life disability may entail creating awareness. Lack of work had a strong link with a disability, though the causality factor cannot be dismissed because an increase in working opportunities can reduce disability among aged individuals.

Effects of risk factors over time are often influenced by effects of age, period (time of data collection), and/or birth cohort. The modeled effects of putative risk factors presented in this report were adjusted for age and period (year of data collection). Strictly speaking, it is not possible to model age, period, and cohort simultaneously [42], because these three characteristics are a linear combination of each other (cohort + age = period). To address this issue, we chose to construct separate models (not shown), adjusting for age and birth cohort. In these, modeled effects of factors other than age, period, and cohort were similar to those in the models reported here. Thus, the effects analyzed were robust with different model specifications. This observation increases confidence that the findings reported here are valid.

## 5. Conclusions

Although overall prevalence has dropped, the absolute number of disabled people continues to increase in Thailand. Thus, the healthcare system and individuals themselves are faced with an increased trouble of disability and new challenges. Since older people who did not work had higher risk for disability in our study, so extending the default retirement age of 60 in Thailand and increasing the work opportunity in older people may reduce the risk of disability although the causality is not clear. In view of the fact that people living alone had a lower risk for disability in our study, so we suppose that older people living alone may be at a lower risk for disability in the future compared to those living with others. However, this needs to be confirmed in future studies.

## Figures and Tables

**Table 1:** The original values of each study variable from each dataset (original variable names are given in bold type).

Dataset		2002	2007	2011

Study year		**Yr**	**Yr**	**Yr**

Age		**A2**	**A2**	**A5**

Marital status		**A5**	**A4**	**A10**

	Original value labels	1 = single	1 = single	1 = single
		2 = married and living together in household	2 = married and living together in household	2 = married and living together in household
		3 = married but not living together in household	3 = married but not living together in household	3 = married but not living together in household
		4 = widowed	4 = widowed	4 = widowed
		5 = divorced	5 = divorced	5 = divorced
		6 = separated	6 = separated	6 = separated

Region		**REG**	**REG**	**REG**

	Original value label	1 = Bangkok	1 = Bangkok	1 = Bangkok
		2 = Central (excluding Bangkok)	2 = Central (excluding Bangkok)	2 = Central (excluding Bangkok)
		3 = North	3 = North	3 = North
		4 = Northeast	4 = Northeast	4 = Northeast
		5 = South	5 = South	5 = South

Area		**Area**	**Area**	**Area**

	Original value label	1 = municipal	1 = municipal	1 = municipal
		2 = nonmunicipal	2 = nonmunicipal	2 = nonmunicipal

Living status		**B12**	**Members**	**Members**

	Original value label	1 = living alone	1 = living alone	1 = living alone
		2 = living with others	2 = living with others	2 = living with others

Working status		**C1**	**A52**	**OP14**

	Original value label	1 = working	1 = working	1 = working
		2 = not working	2 = not working	2 = not working

Income		**C25**	**A80**	**OP41**

	Original value label	0 = no income	1 = more than adequate	1 = more than adequate
		1 = inadequate	2 = adequate	2 = adequate
		2 = adequate	3 = somewhat adequate	3 = somewhat adequate
		3 = more than adequate	4 = inadequate	4 = inadequate
		9 = not known		

Hypertension		**G16**	**B22**	**Op96_1**
Diabetes		**G18**	**B23**	**Op96_2**
Heart disease		**G19**	**B24**	**Op96_3**
Cancer		**G21**	**B25**	**Op96_5**
Stroke		**G26/27**	**B26**	**Op96_13**

	Original value labels	0 = no	0 = no	0 = no
		1 = yes	1 = yes	1 = yes
		2 = not known	2 = not known	2 = not known

Eating		**G33**	**B28**	**Op104**
Dressing		**G34**	**B29**	**Op105**
Squatting		**G36**	**B31**	**Op111**
Lifting 5 kg		**G37**	**B32**	**Op112**
Stairs: 2-3 steps		**G39**	**B34**	**Op114**
Bus/boat alone		**G40**	**B35**	**Op115**

	Original value label	0 = no, cannot do	0 = no	1 = no
		1 = yes, can do	1 = can do with aid	2 = yes, with aid
		9 = not known (considered cannot do)	2 = can do without aid	3 = yes, without aid

**Table 2:** New study variables.

Variables	New value label
Eating	0 = no, can do; 1 = yes, cannot do
Dressing	0 = no, can do; 1 = yes, cannot do
Squatting	0 = no, can do; 1 = yes, cannot do
Lifting 5 kg	0 = no, can do; 1 = yes, cannot do
Stairs: 2-3 steps	0 = no, can do; 1 = yes, cannot do
Bus/boat alone	0 = no, can do; 1 = yes, cannot do
Age (years)	Continuous
Chronic disease	0 = no; 1 = presenting at least one of five chronic diseases (hypertension, diabetes, cancer, stroke, heart)
Education	0 = any education; 1 = no education
Female	0 = no; 1 = yes
Income	0 = adequacy; 1 = inadequacy
Living arrangement	0 = living alone; 1 = living with others
Marital status	0 = never married (reference)
	1 = married, living as couple
	2 = married, not living as couple
Working status	0 = working; 1 = not working
Rural	0 = no; 1 = yes
Bangkok	0 = no; 1 = yes
Central (excluding Bangkok)	0 = no; 1 = yes
North	0 = no; 1 = yes
Northeast	0 = no; 1 = yes
South	0 = no; 1 = yes (reference)
Year 2002	0 = no; 1 = yes (reference)
Year 2007	0 = no; 1 = yes
Year 2011	0 = no; 1 = yes

**Table 3:** Characteristics of study population and unadjusted prevalence of disability, by year, weighted by probability weight.

	2002	2007	2011	Total
	*N* (%)	*N* (%)	*N* (%)	*N* (%)
*N*	24,835	30,427	34,173	89,435
*Demographic*				
Mean age	68.61 ± 7.2	69.03 ± 7.4	69.24 ± 7.5	68.96 (7.3)
Female	13,480 (54.3)	16,859 (55.4)	19,119 (55.9)	49,938 (55.7)
No education	5,113 (20.6)	5,002 (16.4)	4,030 (11.8)	14,145 (16.26)
Low income	8,548 (34.4)	12,751 (41.9)	13,149 (38.6)	34,448 (38.3)
Did not work	16,848 (67.8)	19,552 (64.3)	21,077 (61.7)	57,477 (64.6)
*Marital status*				
Married not together	7,732 (35.2)	10,592 (34.8)	12,352 (36.1)	22,944 (35.4)
Married couple	13,677 (62.2)	19,001 (62.4)	20,494 (60.0)	53,172 (61.5)
Never married	584 (2.7)	834 (2.7)	1,325 (3.9)	2,743 (3.1)
*Living arrangement*				
Living alone	1,554 (6.3)	2,332 (7.7)	2,933 (8.6)	6,819 (7.5)
Living with another	23,281 (93.7)	28,095 (92.3)	31,240 (91.4)	82,616 (92.5)
*Chronic disease*				
Absent	17,482 (70.4)	18,176 (59.7)	19,210 (56.2)	54,868 (62.1)
At least one chronic disease	7,353 (29.6)	12,251 (40.3)	14,963 (43.8)	34,567 (37.9)
*Geographic*				
Rural	17,130 (69.0)	21,737 (71.4)	22,728 (66.5)	61,595 (68.9)
Bangkok	2,577 (10.4)	2,806 (9.2)	3,366 (9.9)	8,749 (9.8)
Central (excluding Bangkok)	6,373 (25.7)	7,166 (23.6)	7,918 (23.2)	21,457 (24.2)
North	5,230 (21.1)	6,360 (20.9)	6,948 (20.3)	18,538 (20.8)
Northeast	7,591 (30.6)	10,224 (33.6)	11,647 (34.1)	29,462 (32.8)
South	3,063 (12.3)	3,872 (12.7)	4,293 (12.6)	11,228 (12.5)
*Prevalence*				
Lifting 5 kg	9,067 (37.3)	8,147 (26.9)	9,970 (29.2)	26,730 (30.7)
Travelling alone	7,019 (30.4)	7,792 (25.8)	8,207 (24.0)	23,018 (26.3)
Climbing 2-3 steps	2,326 (10.1)	4,103 (13.6)	4,066 (11.9)	10,495 (12.0)
Squatting	1,514 (6.5)	3,738 (12.4)	4,325 (12.7)	9,577 (10.9)
Dressing	593 (2.1)	915 (3.0)	926 (2.7)	2,334 (2.7)
Eating	274 (1.2)	684 (2.3)	738 (2.2)	1,696 (1.9)
*Number of disabilities*				
1-2	9,410 (40.7)	8,299 (27.4)	10,154 (29.7)	27,863 (31.8)
≥3	967 (4.2)	2,339 (7.7)	2,313 (6.8)	5,619 (6.4)
Any disability	10,377 (44.9)	10,638 (35.2)	12,467 (36.5)	33,482 (38.4)

**(a):** 

Independent variable	Any disabilities			Eating		
	Coefficient	Odds ratio (95% CI)	*p* value	Coefficient	Odds ratio (95% CI)	*p* value
Age (years)	0.11	1.11 (1.10–1.11)	<0.001	0.09	1.09 (1.08–1.10)	<0.001
Female versus male	0.63	1.88 (1.81–1.94)	<0.001	−0.30	0.74 (0.66–0.83)	<0.001
No education versus any education	0.33	1.39 (1.32–1.45)	<0.001	0.46	1.59 (1.41–1.79)	<0.001
Inadequate versus adequate income	0.18	1.20 (1.16–1.24)	<0.001	0.26	1.29 (1.16–1.43)	<0.001
Married (couple) versus unmarried	−0.21	0.81 (0.74–0.89)	<0.001	−0.60	0.55 (0.42–0.72)	<0.001
Married (not together) versus unmarried	−0.07	0.93 (0.85–1.02)	0.146	−0.53	0.59 (0.45–0.77)	<0.001
Living with others versus alone	0.11	1.12 (1.05–1.19)	<0.001	0.68	1.96 (1.54–2.51)	<0.001
Did not work in the past 7 days	0.97	2.64 (2.54–2.75)	<0.001	1.64	5.15 (4.10–6.47)	<0.001
Any chronic disease^*∗*^	0.57	1.77 (1.71–1.83)	<0.001	1.16	3.19 (2.85–3.56)	<0.001
Rural versus urban	0.02	1.02 (0.98–1.06)	0.316	−0.26	0.77 (0.69–0.87)	<0.001
Bangkok versus South	0.21	1.23 (1.14–1.33)	<0.001	−0.04	0.96 (0.79–1.18)	0.719
Central versus South	0.13	1.14 (1.08–1.21)	<0.001	−0.01	0.99 (0.84–1.16)	0.896
North versus South	0.33	1.39 (1.31–1.48)	<0.001	−0.58	0.56 (0.46–0.67)	<0.001
Northeast versus South	0.34	1.40 (1.32–1.48)	<0.001	−0.27	0.76 (0.64–0.90)	0.002
Year 2007 versus 2002	−0.58	0.56 (0.54–0.58)	<0.001	0.66	1.93 (1.65–2.25)	<0.001
Year 2011 versus 2002	−0.52	0.60 (0.57–0.62)	<0.001	0.56	1.75 (1.50–2.04)	<0.001
Constant	−8.99		<0.001	−12.47		<0.001

**(b):** 

Independent variable	Dressing			Squatting		
	Coefficient	Odds ratio (95% CI)	*p* value	Coefficient	Odds ratio (95% CI)	*p* value
Age (years)	0.09	1.09 (1.09–1.10)	<0.001	0.08	1.08 (1.08–1.09)	<0.001
Female versus male	−0.28	0.76 (0.68–0.83)	<0.001	0.30	1.36 (1.29–1.43)	<0.001
No education versus any education	0.29	1.34 (1.20–1.49)	<0.001	0.12	1.13 (1.06–1.20)	<0.001
Inadequate versus adequate income	0.34	1.41 (1.29–1.54)	<0.001	0.23	1.26 (1.20–1.32)	<0.001
Married (couple) versus unmarried	−0.72	0.49 (0.39–0.62)	<0.001	−0.16	0.85 (0.74–0.97)	0.015
Married (not together) versus unmarried	−0.48	0.62 (0.49–0.78)	<0.001	−0.09	0.92 (0.80–1.05)	0.196
Living with others versus alone	1.03	2.79 (2.21–3.52)	<0.001	0.37	1.45 (1.32–1.59)	<0.001
Did not work in the past 7 days	1.80	6.03 (4.88–7.45)	<0.001	0.96	2.61 (2.44–2.80)	<0.001
Any chronic disease^*∗*^	1.23	3.41 (3.10–3.76)	<0.001	0.73	2.07 (1.97–2.17)	<0.001
Rural versus urban	−0.18	0.83 (0.75–0.93)	0.001	−0.18	0.84 (0.79–0.88)	<0.001
Bangkok versus South	−0.01	0.99 (0.83–1.18)	0.887	0.05	1.05 (0.95–1.16)	0.360
Central versus South	−0.04	0.97 (0.84–1.11)	0.633	0.02	1.02 (0.94–1.10)	0.645
North versus South	−0.34	0.71 (0.61–0.83)	<0.001	−0.06	0.94 (0.87–1.02)	0.165
Northeast versus South	−0.34	0.71 (0.61–0.83)	<0.001	−0.38	0.69 (0.63–0.74)	<0.001
Year 2007 versus 2002	0.33	1.38 (1.28–1.57)	<0.001	0.73	2.07 (1.93–2.22)	<0.001
Year 2011 versus 2002	0.14	1.15 (1.02–1.30)	0.023	0.72	2.05 (1.91–2.19)	<0.001
Constant	−12.53		<0.001	−9.65		<0.001

**(c):** 

Independent variable	Lifting 5 kg			Climbing 2-3 steps		
	Coefficient	Odds ratio (95% CI)	*p* value	Coefficient	Odds ratio (95% CI)	*p* value
Age (years)	0.09	1.10 (1.09–1.10)	<0.001	0.10	1.10 (1.09–1.10)	<0.001
Female versus male	0.62	1.85 (1.79–1.92)	<0.001	0.22	1.25 (1.19–1.32)	<0.001
No education versus any education	0.24	1.27 (1.21–1.33)	<0.001	0.19	1.21 (1.14–1.28)	<0.001
Inadequate versus adequate income	0.09	1.09 (1.05–1.13)	<0.001	0.30	1.35 (1.29–1.41)	<0.001
Married (couple) versus unmarried	−0.08	0.93 (0.84–1.02)	0.115	−0.41	0.66 (0.58–0.76)	<0.001
Married (not together) versus unmarried	0.06	1.06 (0.97–1.17)	0.214	−0.24	0.79 (0.69–0.89)	<0.001
Living with others versus alone	0.09	1.10 (1.03–1.17)	0.004	0.32	1.38 (1.27–1.51)	<0.001
Did not work in the past 7 days	0.97	2.64 (2.53–2.76)	<0.001	1.39	4.04 (3.73–4.37)	<0.001
Any chronic disease^*∗*^	0.53	1.70 (1.64–1.76)	<0.001	0.77	2.16 (2.06–2.26)	<0.001
Rural versus urban	−0.08	0.92 (0.89–0.96)	<0.001	−0.04	0.96 (0.91–1.01)	0.126
Bangkok versus South	0.30	1.35 (1.25–1.45)	<0.001	−0.25	0.78 (0.70–0.86)	<0.001
Central versus South	0.05	1.05 (0.99–1.11)	0.138	−0.19	0.83 (0.77–0.89)	<0.001
North versus South	0.35	1.42 (1.33–1.51)	<0.001	−0.42	0.66 (0.61–0.72)	<0.001
Northeast versus South	0.35	1.42 (1.34–1.51)	<0.001	−0.16	0.85 (0.79–0.92)	<0.001
Year 2007 versus 2002	−0.64	0.53 (0.50–0.55)	<0.001	0.31	1.37 (1.28–1.45)	<0.001
Year 2011 versus 2002	−0.53	0.59 (0.57–0.62)	<0.001	0.09	1.09 (1.02–1.16)	0.007
Constant	−8.62		<0.001	−10.50		<0.001

**(d):** 

Independent variable	Travelling alone		
	Coefficient	Odds ratio (95% CI)	*p* value
Age (years)	0.12	1.12 (1.12–1.13)	<0.001
Female versus male	0.49	1.64 (1.57–1.71)	<0.001
No education versus any education	0.35	1.42 (1.36–1.49)	<0.001
Inadequate versus adequate income	0.25	1.28 (1.23–1.33)	<0.001
Married (couple) versus unmarried	−0.33	0.72 (0.65–0.80)	<0.001
Married (not together) versus unmarried	−0.18	0.83 (0.75–0.92)	<0.001
Living with others versus alone	0.16	1.18 (1.10–1.26)	<0.001
Did not work in the past 7 days	1.19	3.28 (3.12–3.44)	<0.001
Any chronic disease^*∗*^	0.61	1.85 (1.78–1.92)	<0.001
Rural versus urban	0.12	1.13 (1.08–1.18)	<0.001
Bangkok versus South	0.10	1.11 (1.02–1.21)	0.014
Central versus South	0.08	1.08 (1.01–1.15)	0.019
North versus South	0.08	1.08 (1.01–1.15)	0.020
Northeast versus South	0.20	1.22 (1.15–1.30)	<0.001
Year 2007 versus 2002	−0.37	0.69 (0.66–0.72)	<0.001
Year 2011 versus 2002	−0.51	0.60 (0.57–0.63)	<0.001
Constant	−10.51		<0.001

^*∗*^Any chronic disease includes heart problems, hypertension, diabetes, cancer, and stroke.

**Table 5:** Proportional impacts of independent variables on prevalence of six disabilities in the elderly in Thailand in 2002, 2007, and 2011.

Independent variable	Having difficulty with indicated activity						Overall average
	Lifting 5 kg	Traveling by oneself	Climbing stairs	Squatting	Dressing	Eating	
Did not work for 7 days	0.560	0.647	0.685	0.545	0.600	0.574	0.602
Age 65 to 75	0.608	0.739	0.586	0.532	0.374	0.384	0.537
Any of 5 chronic diseases^*∗*^	0.338	0.383	0.462	0.489	0.559	0.546	0.463
Lives with others versus lives alone	0.057	0.094	0.160	0.202	0.264	0.205	0.164
No formal education versus any education	0.154	0.227	0.111	0.077	0.122	0.215	0.151
Lower versus higher income	0.053	0.149	0.170	0.148	0.135	0.104	0.126
Female versus male	0.376	0.292	0.122	0.187	−0.108	−0.122	0.125
Year 2007 versus 2002	−0.380	−0.216	0.179	0.501	0.129	0.290	0.084
Year 2011 versus 2002	−0.318	−0.298	0.048	0.480	0.055	0.237	0.034
Bangkok versus South	0.195	0.064	−0.127	0.029	−0.005	−0.014	0.024
Central versus South	0.029	0.046	−0.101	0.011	−0.013	−0.004	−0.005
Northeast versus South	0.224	0.124	−0.087	−0.224	−0.121	−0.103	−0.031
North versus South	0.227	0.047	−0.207	−0.035	−0.116	−0.198	−0.047
Rural versus urban	−0.049	0.071	−0.024	−0.116	−0.071	−0.107	−0.050
Married, not living as couple, versus unmarried	0.038	−0.109	−0.129	−0.053	−0.171	−0.197	−0.104
Married, living as couple, versus unmarried	−0.049	−0.204	−0.237	−0.105	−0.300	−0.260	−0.192

^*∗*^Any chronic diseaseincludes heart problems, hypertension, diabetes, cancer, and stroke.
